# Editorial: Advances in methods of biochemical assessment and diagnosis of animal welfare in wildlife

**DOI:** 10.3389/fvets.2025.1602735

**Published:** 2025-04-16

**Authors:** Ana María Molina-López, Valeria Pasciu, Elena Baralla, Guadalupe Gómez-Baena

**Affiliations:** ^1^Departamento de Anatomía y Anatomía Patológica Comparadas, y Toxicología, Universidad de Córdoba, Cordoba, Spain; ^2^Department of Veterinary Medicine, University of Sassari, Sassari, Italy; ^3^Departamento de Bioquímica y Biología Molecular, Universidad de Córdoba, Cordoba, Spain

**Keywords:** welfare, wildlife, hormone levels, physiological status, gut microbiota

**Figure 1:**
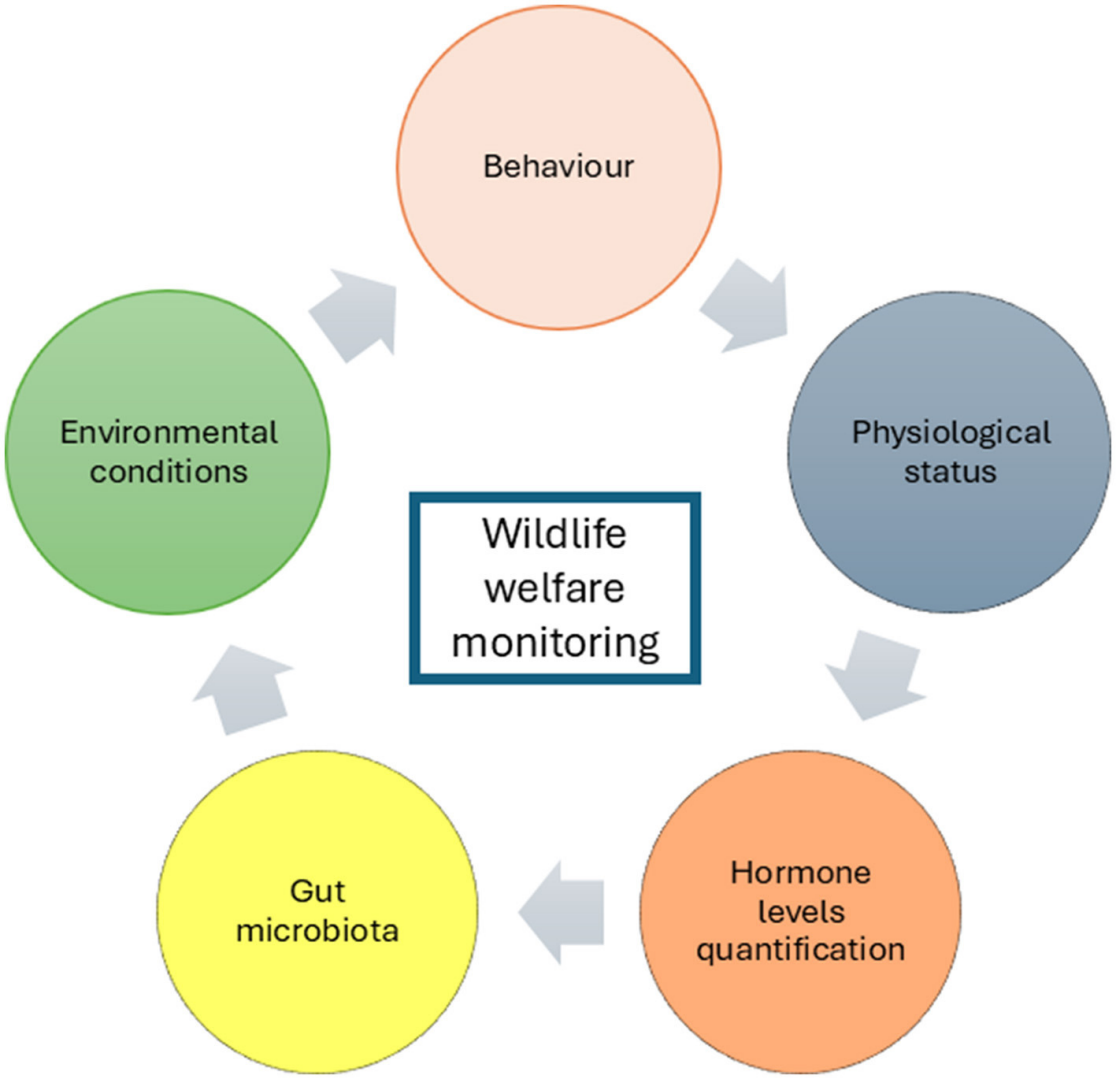
Main parameters for monitoring animal welfare in wildlife.

Factors contributing to stress or the application of deficient methods to ensure animal welfare could negatively affect various aspects of animal physiology. This is especially significant for endangered species, as it may increase their susceptibility to zoonotic diseases, which can have a direct impact on both public health and biodiversity conservation (Sonnega and Sheriff). This Research Topic aims to compile articles on the latest scientific advances in the development and validation of new diagnostic methods for the early identification of biomarkers, which will be determinant in predicting early alterations in animal welfare (Figure 1).

Thyroid hormones (THs) are related to the endocrine regulation of body temperature and can be considered indicators of animals' metabolic and nutritional state (Pasciu et al.). THs, like others such as cortisol, can be affected by environmental conditions, so, monitoring them during different times of the year will provide relevant information (Pasciu et al.; Supanta et al.). Pasciu et al. conducted a review on the TH metabolites in feces (FTMs) of wild ungulates, examining both the different methods used for their assay and their fluctuations related to individual or environmental variables, to identify common trends between species. These Authors found that FTM levels were higher in colder periods, decreasing with age, and were unrelated to sex, food quality, animal behavior, anthropogenic disturbance, and body condition. Furthermore, FTMs were high especially in developing animals, confirming the importance of TH levels in individual growth. The importance of validating analytical techniques in each matrix and animal species was also emphasized. Authors concluded that non-invasive assays for determining FTMs can represent a promising tool to evaluate animal response to environmental modifications and their adaptive capacity.

The effect of isolation on the welfare of captive Asian elephants (*Elephas maximus*) has been studied. Supanta et al. assigned a body condition score to each animal, and blood samples were taken to analyze muscle enzymes, liver enzymes, lipids, and metabolic function. The study confirmed that during this period the animals' physical activity reduced, decreasing the creatine kinase (CK) and increasing the aspartate aminotransferase and alkaline phosphatase. CK is associated with cellular activity, exercise, muscle damage, or trauma. Additionally, the decrease in the intake of bananas and sugar cane was associated with a reduction in triglyceride levels and a lower number of animals in the obese category. In a subsequent study, Supanta et al. took blood and fecal samples to measure fecal glucocorticoid metabolites (FGCM) and to evaluate oxidative stress in serum through the determination of malondialdehyde (MDA) and 8-hydroxy -deoxyguanosine (8-OHdG), along with stress leukograms. They observed an increase in FGCM levels, as well as the heterophil/lymphocyte (H/L) ratio, a measure affected by cortisol. Serum 8-OHdG, an indicator of DNA oxidative damage, also increased over time, while monocytosis and lymphopenia further suggested alterations in immune function due to stress. In contrast, serum MDA declined, possibly in response to reduced roughage and supplement intake. These results highlighted the need of implementing contingency plans to improve the management of elephant enclosures, such as maintaining adequate physical activity, ensuring that the health and welfare needs of these animals are met when there is any interference in the tourism industry.

Seabird species could serve as sentinels of marine ecosystems and establishing baseline clinical parameters could provide useful information to apply in the conservation and recovery of wildlife. For example, in the marine environment, acute phase proteins (APP) and haptoglobin (HP) can provide information about the health of marine mammals exposed to oil or other contaminants. Therefore, Lee et al. established baseline reference parameters for serum protein electrophoresis, APP including serum amyloid A (SAA) and HP, and biochemistry parameters in the Rhinoceros auklet (*Cerorhinca monocerata*). SAA and HP, among other biomarkers, are used to monitor inflammation in birds. These authors indicate that SAA could be an indicator of inflammation and potential subclinical diseases. They did not observe sex differences in the plasma values studied. When comparing plasma triglyceride levels with other species, it has been observed that Rhinoceros auklet shows higher levels during egg-laying and breeding season, probably due to dietary differences between species.

Gut microbiome has emerged as a novel biomarker for assessing animal welfare. Its composition and relationship with the host can reflect health status, stress levels, and emotional states. According to the review by Sonnega and Sheriff, techniques for assessing behavior in wild animals are becoming essential tools for evaluating welfare. Indicators such as activity levels, vocalizations, and aggression offer valuable insights into the emotional and physical states of these animals. However, to ensure their accuracy, behavioral metrics need to be validated using other welfare-related measures, such as stress physiology. The gut microbiome provides a wealth of information about physiology (energy metabolism, thermal regulation, fat deposition, immune function, hormonal regulation) and its influence on host behavior.

Beale et al. employed a multi-omics approach in feces to assess stress in farmed saltwater crocodiles (*Crocodylus porosus*). They explored the relationship between glucocorticoids, metabolites, and gut microbiota, in a non-invasive manner. The authors found no significant differences in fecal glucocorticoid levels in crocodiles housed individually or in groups. However, metabolic profiling unveiled unique stress responses: crocodiles housed individually exhibited alterations in the levels of specific compounds, which influenced pyrimidine and purine metabolic pathways associated with stress related to energy demand, in comparison to those housed in groups. Furthermore, analysis of the fecal microbiome suggested elevated stress levels in group-housed crocodiles, potentially driven by dominance hierarchies that could induce anxiety in subordinate animals.

In summary, the previously mentioned reports highlight the importance of conducting a comprehensive assessment of animal welfare, taking into account behavioral, physiological, and environmental parameters such as housing conditions, weather circumstances, food availability, or environmental pollution, among others. Omics techniques are positioned as key tools in the identification of new biomarkers. It is essential to use validated techniques for each species, preferably non-invasive methods, and to incorporate novel biomarkers for welfare evaluation, to ensure correct interpretation and application in species conservation programs.

